# Effect of Abutment Material on aMMP-8 Levels in Peri-Implant Sulcular Fluid over 12 Months: A Randomized Controlled Trial

**DOI:** 10.3390/diagnostics15030264

**Published:** 2025-01-23

**Authors:** Behrouz Arefnia, Kerstin Theisen, Elisabeth Steyer, Martin Lorenzoni, Armin Sokolowski, Ceeneena Ubaidha Maheen, Taina Tervahartiala, Timo Sorsa, Alwin Sokolowski

**Affiliations:** 1 Division of Restorative Dentistry, Periodontology and Prosthodontics, Department of Dental Medicine and Oral Health, Medical University of Graz, 8010 Graz, Austria; behrouz.arefnia@medunigraz.at (B.A.);; 2 Department of Oral and Maxillofacial Diseases, Institute of Dentistry, Helsinki University Central Hospital (HUCH), University of Helsinki, 00014 Helsinki, Finland

**Keywords:** peri-implant inflammation, matrix metalloproteinase-8 (MMP-8), abutment materials, titanium, zirconium oxide, polymethylmethacrylate (PMMA), peri-implant sulcular fluid, dental implants, biomaterials, peri-implant health

## Abstract

**Background/Objectives:** The long-term success of dental implants can be influenced by the material properties of abutments and their interaction with peri-implant tissues. This study investigates the impact of three abutment materials—titanium (Ti), zirconium oxide (Zr), and polymethylmethacrylate (PMMA)—on the inflammatory response in peri-implant sulcular fluid (PISF), using active-matrix metalloproteinase-8 (aMMP-8) as a biomarker. **Methods:** In this prospective, randomized clinical trial, 30 patients were assigned to Ti, Zr, or PMMA abutment groups. PISF samples were collected at predefined intervals over 12 months and analyzed for aMMP-8 levels using enzyme-linked immunosorbent assays (ELISA). Clinical parameters (probing depth, bleeding on probing, and plaque index) and radiographic assessments of bone resorption were also evaluated. **Results:** Two weeks after implant uncovering, baseline aMMP-8 levels varied significantly among materials, with Zr demonstrating the highest levels. Over time (2, 3, 6 and 12 months after implant uncovering), aMMP-8 levels decreased across all groups, with no significant differences observed at 12 months. Radiographic assessments indicated no statistically significant differences in bone resorption, with clinical parameters remaining comparable across all groups. **Conclusions:** Initial inflammatory responses to abutment materials may vary; however, all tested materials—Ti, Zr, and PMMA—showed long-term biocompatibility and supported healthy peri-implant tissue integration. These findings indicate that selecting any of the tested abutment materials does not significantly affect long-term peri-implant health.

## 1. Introduction

The success of dental implants depends not only on the quality and quantity of supporting bone but also on the integration of implant materials with adjacent soft tissues [1]. Plaque accumulation on rough or retentive surfaces and materials that irritate surrounding tissues can provoke inflammatory responses, leading to complications such as chronic inflammation, tissue degradation, hypersensitivity reactions, and potentially implant failure [2]. This can compromise osseointegration and increase the risk of peri-implantitis, further jeopardizing long-term stability and success.

Modifying the surfaces of abutments is a key strategy to mitigate peri-implantitis, as surface characteristics like roughness and chemical composition can significantly influence plaque formation [3,4]. The initial adhesion and subsequent colonization of bacteria on surfaces that are in constant contact with soft tissues are critical in the onset of biomaterial-related infections [5].

A wide range of materials and surface treatments have been extensively investigated with the aim of reducing dysbiotic bacterial plaque development on implant surfaces and implant abutments [6]. Various surface modifications, including smooth, polished surfaces, hydrophilic coatings, and antimicrobial treatments, as well as materials such as titanium, zirconia, and polymer composites, have been extensively explored [7]. These materials and treatments have been tested in the oral cavity to assess their potential effectiveness in minimizing bacterial adhesion and biofilm formation. These efforts aim to enhance long-term clinical outcomes by improving tissue integration and reducing the risk of peri-implant diseases. However, further clinical research is required to refine these approaches and address remaining gaps in understanding [8,9,10,11,12]. Inflammatory parameters around implants are essential for describing disease progression [13]. However, some patients naturally exhibit higher baseline inflammation levels, for example, in the presence of pre-existing periodontal or local inflammation [14]. Specific genotypes and systemic conditions, such as diabetes or smoking, can also exhibit an increased inflammatory response to foreign materials in the oral cavity, such as zirconia, metals, or other restorative materials [15,16,17].

Matrix metalloproteinases (MMPs), particularly MMP-8, play a crucial role in the breakdown of the extracellular matrix, tissue remodeling, and disease progression. As identified by DeCarlo et al. (1998) and Sorsa et al. (2006, 2010), MMP-8 is a key mediator in the destruction of periodontal and peri-implant tissues, making it an essential biomarker for monitoring disease progression [18,19,20]. Previous research has established active MMP-8 (aMMP-8) as a reliable indicator for both peri-implant and periodontal diseases, with low aMMP-8 levels typically indicating a healthy state in cases of periodontitis [21,22]. Elevated aMMP-8 levels are strongly associated with tissue degradation and inflammation, distinguishing it from non-collagenolytic latent MMP-8, thereby making it a valuable tool for early diagnosis and disease management [23,24]. It is important not to equate aMMP-8 with MMP-8 in the diagnostics of peri-implantitis and periodontitis [25].

Studies have shown that effective peri-implant treatments, such as antimicrobial therapy, debridement, and the use of anti-inflammatory agents, significantly reduce aMMP-8 levels, correlating with reduced inflammation and improved clinical outcomes [23,26,27]. Intrasulcular levels of aMMP-8 have been shown to significantly influence the inflammatory status of peri-implant tissues. Regular maintenance therapy is essential for long-term implant success, as it involves thorough decontamination of prosthetic components and surrounding tissues to prevent bacterial colonization and manage inflammatory responses, thus helping to control aMMP-8 levels. Collectively, these findings underscore the critical role of aMMP-8 in understanding, diagnosing, and managing peri-implant diseases, offering valuable insights for enhancing clinical practice and improving patient care outcomes. Monitoring aMMP-8 levels in peri-implant sulcus fluid (PISF) could aid in the early diagnosis of mucositis and peri-implantitis even before clinical manifestations appear, allowing for timely intervention and the initiation of appropriate therapy.

The present study investigates the inflammatory response in PISF adjacent to abutments made from polymethylmethacrylate (PMMA), titanium (Ti), and zirconium oxide (Zr). It aims to evaluate the impact of these materials on peri-implant disease through clinical and biomarker analyses, with a focus on aMMP-8 levels in PISF. By examining the effects of PMMA, Zr, and Ti abutments, the study seeks to understand the influence of abutment material on peri-implant inflammatory responses over time within a prospective controlled study design.

## 2. Materials and Methods

This prospective, controlled, randomized clinical trial was conducted over 15 months and included 30 patients. Eligible volunteers were randomly assigned before implant placement to one of three groups: Group 1 received titanium (Ti) abutments, Group 2 received zirconium oxide (Zr) abutments, and Group 3 received polymethylmethacrylate (PMMA) abutments.

The study was conducted at the Medical University of Graz, with participation being voluntary. Informed consent was obtained from all participants, who received detailed explanations about the study’s purpose, procedures, potential risks, benefits, and possible discomforts. Participants were informed of their right to withdraw at any time without affecting their subsequent medical treatment. Additionally, it was clarified that their medical records might be accessed by authorized investigators not directly involved in their care. All study protocols, patient information sheets, and consent forms were reviewed and approved by the Institutional Ethics Committee (EK 27-188 ex 14/15).

The study was conducted in accordance with Good Clinical Practice (GCP) guidelines, the Declaration of Helsinki, and relevant Austrian laws, including the Medical Devices Act (MPG) and the European Directive on implantable medical devices (90/385/EEC). Compliance with EN-ISO standards was maintained throughout the study.

### 2.1. Patient Selection

Patients were selected for the study based on the following inclusion criteria: the ability to provide informed consent; good general health as determined by medical history; age between 18 and 65 years; good periodontal status (Bleeding on Probing (BOP) < 10%, Full Mouth Plaque Score (FMPS) < 20%, Probing Depth (PD) < 4 mm); non-smoking status; a missing tooth in the posterior maxilla or mandible requiring implant therapy; and bone quality III or IV and bone quantity A or B, according to Lekholm and Zarb (1985) [28]. Exclusion criteria included insufficient bone volume requiring sinus floor elevation or bone augmentation, smoking, uncontrolled diabetes, medications contraindicated for implant therapy (including intake of antibiotics during the study or chemotherapy for malignant conditions), skeletal immaturity, active malignancy or infection at the operative site, hypersensitivity or allergy to any study materials, and pregnancy.

A total of 30 patients requiring implant rehabilitation in the posterior region were enrolled based on these criteria. Recruitment took place at the Division of Prosthodontics, Restorative Dentistry, Periodontology, and Implantology at the Medical University of Graz. Routine preoperative clinical and radiographic assessments, including panoramic x-rays and cone beam computed tomography, were performed. Randomization into the three treatment groups (Ti, Zr, PMMA) was conducted using a web-based randomization tool [29].

### 2.2. Surgical Procedure, Follow-Ups and aMMP-8 Monitoring

All surgeries were performed by a single experienced surgeon (ML) under local anesthesia, adhering to standard aseptic protocols. A 0.2% chlorhexidine digluconate rinse was administered preoperatively. The surgical procedures included mucoperiosteal flap elevation and bone assessment. CE-certified implants (SPI^®^ELEMENT Implantat INICELL^®^, Thommen Medical AG, 2540 Grenchen, Switzerland) were placed pericrestally following the manufacturer’s surgical protocol, ensuring sufficient volume of hard and soft tissue. The implants were left to heal submucosally after applying closure screws and suturing the flap over the implants. A postoperative panoramic X-ray was taken to confirm correct implant placement. Sutures were removed seven days post-surgery. Anti-inflammatory analgesics (Dexibuprofen 400 mg every 8 h) were prescribed and patients were instructed to take them in case of pain or discomfort. No local or systemic antibiotics were prescribed for the perioperative surgical phase.

All implants were equipped with the same uniform internal hexagon prosthetic connection. Regular clinical follow-ups included evaluations of oral hygiene status, and consistent oral hygiene instructions were provided to all patients at each visit to ensure uniform guidance. However, no professional cleaning was performed during the study to avoid influencing outcomes.

**Figure 1:**
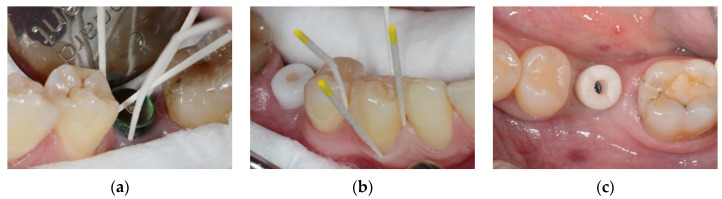
Intraoral photographs of the three groups: (**a**) Ti group with peri-implant sulcus fluid (PISF) collection using paper points at the implant site; (**b**) Zr group with GCF collection using paper points at a tooth site; (**c**) PMMA group showing the PMMA healing abutment in place.

After the healing period, implants were uncovered during a second-stage surgery, and healing abutments made of Ti, Zr, or PMMA were placed according to group assignments. Figure 1 shows the three different materials of healing abutments.

For the PMMA group, prefabricated customizable gingiva formers with a titanium base and PMMA healing abutments were utilized (Thommen Medical AG, Grenchen, Switzerland). The titanium group received sterile prefabricated gingiva formers, while the zirconia group was provided with customizable zirconia grinding abutments (ART grinding cylinder abutment, Thommen Medical AG, Grenchen, Switzerland), which were individually adjusted in height by a dental technician prior to placement.

The primary outcome of interest was the measurement of aMMP-8 levels in PISF, assessed at two weeks, two months, three months, six months, and twelve months after abutment placement. PISF was collected using paper points that were inserted into the peri-implant sulcus for 30 s to absorb crevicular fluid (Figure 1a). Gingival crevicular fluid (GCF) from a neighboring healthy tooth was also collected for comparison (Figure 1b). The natural control tooth chosen for comparison was located in the same quadrant as the implant but was not directly adjacent to it. This tooth was free from endodontic treatment, caries or any restoration reaching the gingival margin.

Collected samples were stored in sterile tubes at −80 °C to preserve enzyme activity and were carefully transported to the laboratory for further analysis. The aMMP-8 levels were quantified using enzyme-linked immunosorbent assay (ELISA) technology. This method utilizes specific monoclonal antibodies that bind exclusively to aMMP-8. The detection process involves a sandwich-based immunoassay technique, wherein antibodies are immobilized on a solid phase, capturing the target aMMP-8. A secondary enzyme-linked antibody is added, and its interaction with the antigen produces a measurable signal, either colorimetric or fluorescent, proportional to the aMMP-8 concentration [20]. The ELISA analysis was conducted by Dentognostics GmbH (Jena, Germany) in accordance with established protocols. This technique has been validated for its reliability and specificity in detecting aMMP-8, as described in previous studies, including those comparing various laboratory methods and chair-side diagnostics for periodontal and peri-implant health assessment [20]. Clinical peri-implant parameters, including PD and BOP, were also assessed using a periodontal probe. Implant stability was assessed using Periotest values (Periotest M, Medizintechnik Gulden, Modautal, Germany) immediately after implant placement and three months postoperatively during re-entry surgery. Soft tissue assessments, including evaluations of Plaque Index (PI), PD and BOP, were performed by a single, calibrated examiner who had been trained and instructed in conducting clinical studies.

Single implant prosthetic restorations were initiated after the third aMMP-8 measurement, three months following re-entry surgery. Patients in the Ti and Zr groups had their final restorations completed at this stage. The Ti group received individual titanium abutments extending to the gingival margin, while the Zr group was provided with zirconia crowns starting from the implant shoulder, ensuring that the respective group characteristics were maintained, since the abutment material had the same surface modifications and identical material characteristics according to the manufacturing company. It was also ensured that no stain or glazing was applied in areas in contact with the mucosa. Patients with PMMA abutments were provided with temporary PMMA restorations. The final aMMP-8 measurement was performed 15 months after implant placement, providing data on the long-term effects of the different abutment materials on peri-implant tissues. Table 1 summarizes the schedule for clinical activities, including radiographic evaluations, implant placement and uncovering, Periotest measurements, and aMMP-8 monitoring, conducted at key timepoints during the study period.

### 2.3. Statistical Considerations

This study included 30 patients, with provisions to replace any participants who dropped out to maintain the sample size. All statistical operations were carried out using statistical software (SPSS, version 27; IBM, Armonk, NY, USA) and spreadsheet software (Excel, version 2016; Microsoft, Redmond, WA, USA).

A power analysis was conducted to determine the appropriate sample size for the study, based on data from a previous split-mouth study with two groups (*n* = 12 per group) investigating the influence of Zr and Ti abutments in peri-implant soft-tissue healing using matrix metalloproteinase-8 [30]. The analysis evaluated the statistical power for detecting significant differences in aMMP-8 levels using both ANOVA and paired *t*-tests. For ANOVA with *p* = 0.011 and an assumed standard deviation (SD) of 2.5, the power was 95%. Similarly, for paired *t*-tests with *p* = 0.018 and a mean difference of −4.05, the power was 95% with SD = 3.0.

Assuming an SD of 2.5, the power was calculated to be 95% for two groups with *n* = 12 per group, indicating a high probability of detecting significant differences. For the present study, which included three groups, the decision was made to include *n* = 10 per group. This was based on the need to balance statistical robustness with feasibility in terms of recruitment, time, and resources. While slightly reducing the sample size, *n* = 10 per group ensures sufficient power for reliable statistical comparisons and allows the study to remain practical within the clinical research framework. The number of participants was considered to provide insights into the effects of different abutment materials on peri-implant inflammatory responses and aMMP-8 levels. The findings from this study will serve as a foundational basis for designing future larger-scale, prospective, multicenter clinical trials.

The null hypothesis is that there is no significant difference in aMMP-8 levels in peri-implant sulcus fluid between titanium, zirconium oxide, and polymethylmethacrylate abutments. Therefore, all three materials are expected to exhibit the same effects on peri- implant tissue reaction.

Data collected were analyzed using one-way ANOVA and a paired *t*-test to evaluate potential differences in aMMP-8 levels across the groups and timepoints.

## 3. Results

A total of 30 patients requiring implant rehabilitation in the posterior region were included in the study. All patients were successfully implanted and assessed, with no implant loss or patient dropout throughout the study period. No technical complications were observed during any of the visits across all groups. Each patient had 11 visits with evaluations conducted at specified timepoints (Table 1). No significant correlations were found between MBL, aMMP-8 levels, or other clinical parameters with respect to age or gender.

Periotest values (PTVs) were assessed at three timepoints using healing abutments: at implant placement, at uncovering, and 15 months after implant placement. Throughout the study, PTVs remained stable, and no significant differences were observed between the Ti, Zr, and PMMA groups. At implant placement, the mean PTVs were −7.9 (±0.34) for the Ti group, −7.8 (±0.46) for the Zr group, and −7.7 (±0.38) for PMMA. At uncovering, the values slightly decreased to −6.9 (±0.51) for Ti, −7.1 (±0.62) for Zr, and −7.0 (±0.57) for PMMA. At 15 months after implant placement, the mean PTVs were −7.9 (±0.48) for Ti, −7.8 (±0.55) for Zr, and −7.9 (±0.50) for PMMA.

At baseline (2 weeks after re-entry surgery), the mean aMMP-8 levels for the sampled implant sites (*N* = 30) were 168.0 ng/mL (±199.6) for Ti, 427.3 ng/mL (±220.6) for Zr, and 287.5 ng/mL (±234.3) for PMMA (Table 2).

In comparison, the corresponding healthy teeth showed mean levels of 299.9 ng/mL (±193.2) for titanium, 279.8 ng/mL (±246.2) for zirconia, and 270.2 ng/mL (±205.9) for PMMA. This indicates that natural teeth generally exhibited higher aMMP-8 levels than their associated implants at baseline.

Differences between the aMMP-8 levels of PISF in implants and GCF of natural teeth are listed in Table 3. When comparing natural teeth with implants within the same patient (*N* = 10 per group), no statistically significant differences were observed due to high variability and the small sample size. However, when the differences (Diff. = Natural tooth − Implant) were analyzed using a paired *t*-test, a significant difference was found at the 2-week timepoint in the zirconia group, whereas no significant differences were observed in the titanium and PMMA groups (Table 3).

A further significant difference was observed 3 months after re-entry surgery, where the Ti group showed a statistically significant reduction in aMMP-8 levels compared to the corresponding natural teeth. No significant differences were noted in the zirconia or PMMA groups at this timepoint.

**Figure 2:**
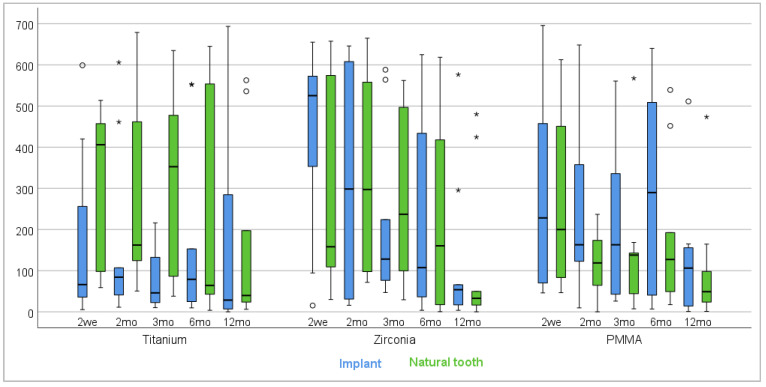
aMMP-8 levels in implants and natural teeth across timepoints (2 weeks to 12 months) for titanium, zirconia, and PMMA abutments. Blue boxes represent implant values, and green boxes represent natural tooth values. Mild outliers indicated by a circle (°); extreme outliers indicated by an asterisk (*).

By the 12-month follow-up, aMMP-8 levels had decreased across all groups, with reduced variability between the materials, suggesting successful healing and minimal peri-implant inflammation (Figure 2). However, no statistically significant differences were observed between the materials. When analyzed using one-way ANOVA across all timepoints (2 weeks, 2 months, 3 months, 6 months, and 12 months), no significant differences in aMMP-8 levels were found between the three material groups. The only significant difference was observed at the baseline measurement, where the Zr group showed the highest aMMP-8 levels.

**Figure 3:**
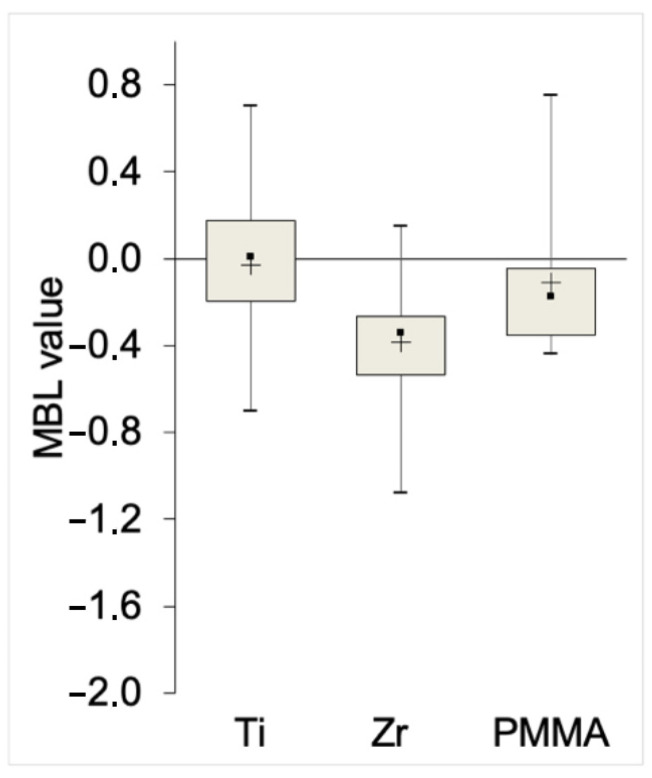
Mean marginal bone level (MBL) values at 12 months after uncovering for Ti, Zr and PMMA abutments. The plot shows mean values with standard deviations (SD) and ranges (min to max). Ti exhibited the least bone resorption, followed by PMMA and Zr.

Radiographic measurements of the marginal bone level (MBL) using the right-angle technique, taken 12 months after uncovering, revealed slightly greater bone loss in Zr compared to PMMA and Ti, with Ti exhibiting the least bone loss. The measured MBL at 12 months was −0.03 mm (±0.37 mm) for Ti, −0.38 mm (±0.33 mm) for Zr, and −0.11 mm (±0.33 mm) for PMMA (Figure 3).

**Figure 4:**
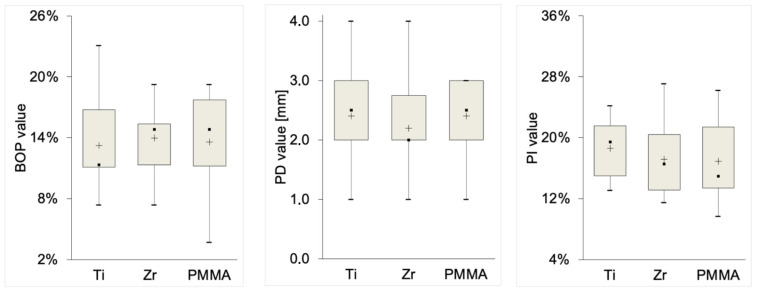
Clinical parameters 12 months after uncovering across the three groups: bleeding on probing (BOP), probing depth (PD), and plaque index (PI). No significant differences were observed between Ti, Zr, and PMMA groups for BOP and PD over time. The PI remained stable across all timepoints.

Similarly, clinical parameters such as BOP and PD did not show significant differences between the groups 12 months after re-entry surgery (Figure 4). The PI remained stable across all timepoints.

In summary, no significant differences in aMMP-8 levels, MBL, or clinical parameters were observed between the Ti, Zr, and PMMA groups over the 12-month follow-up period. All three materials exhibited high biocompatibility, effective osseointegration, and healthy peri-implant tissue responses.

## 4. Discussion

The present study investigated the influence of abutment materials—Ti, Zr, and PMMA—on the levels of aMMP-8 in PISF. aMMP-8 is a well-established biomarker for peri-implant inflammation and tissue degradation, making it a useful tool for assessing peri-implant health [31]. These findings contribute to a growing body of evidence on how biomaterials influence peri-implant tissue responses, with a particular focus on inflammatory markers in peri-implant soft tissues.

The analysis revealed a significant difference in aMMP-8 levels between Zr abutments and natural teeth at baseline, whereas no significant differences were found for Ti or PMMA. These results are of interest, as previous findings suggest that the immunological response in peri-implant soft tissues adjacent to Ti abutments was less favourable compared to Zr or coated zirconium oxide abutments [32,33]. The biological mechanisms behind this could be related to surface characteristics, potentially triggering an early immune response.

In a comprehensive review, Alarcón-Sánchez et al. (2023) stated that metal-free prosthetic materials may induce a lower inflammatory response compared to metal-based ones, possibly due to changes in the subgingival microbiota that lead to dysbiosis and a higher prevalence of pathogenic bacteria [34]. However, this heightened response did not persist, as subsequent aMMP-8 measurements showed no significant differences between the materials at later timepoints. Interestingly, natural teeth, which served as a reference for each patient, consistently displayed higher aMMP-8 levels compared to their corresponding implants. This comparison to each patient’s healthy teeth was critical, as completely healthy patients typically have low baseline aMMP-8 levels. However, older patients or those with prior inflammation might naturally have elevated aMMP-8 levels. Therefore, using each patient’s natural tooth as an internal control allowed us to account for individual variability in baseline inflammation levels and better assess the material’s effect on peri-implant tissues.

At the 3-month follow-up, a significant reduction in aMMP-8 levels was observed in Ti abutments compared to natural teeth. No significant differences were detected in the Zr or PMMA groups at this timepoint in comparison to natural teeth.

By the 12-month visit, aMMP-8 levels had decreased across all groups, with reduced variability between the materials. Regardless of the initial response, peri-implant tissues eventually stabilized, as evidenced by the absence of significant differences between the groups at this timepoint. This suggests that all three materials (Ti, Zr, and PMMA) support long-term peri-implant health, as indicated by low aMMP-8 levels and the clinical absence of inflammation as low aMMP-8 in PISF, GCF and mouth-rinse have been associated with peri-implant and periodontal health [35].

In addition to aMMP-8 levels, radiographic MBL and clinical parameters, including BOP, PD, and PI, were evaluated. The radiographic data revealed slightly more bone loss with Zr implants compared to PMMA and Ti, with Ti showing the least bone loss. However, these differences were not statistically significant. Similarly, no significant differences were found in clinical parameters across the groups. These results reinforce the idea that while initial differences in inflammatory markers may exist between the three different abutment materials, these differences do not translate into clinically significant changes in peri-implant tissue health over time.

In contrast to other studies that found higher levels of MMP-8 in patients with metal restorations and concomitant generalized periodontitis, we had no patients with active periodontal disease, as this could result in possibly higher aMMP-8 levels at the natural tooth site.

These findings align with prior research that highlights the similar biological performance of different abutment materials over time. While Kumar et al. (2017) observed that Ti abutments exhibited significantly higher MMP-8 levels and probing depths than zirconia abutments one and three months after reentry, there were no significant differences after 12 months. This corresponds with the present results, which also showed a general decrease in aMMP-8 levels across all groups over time, suggesting that early differences in inflammatory responses between materials diminish as healing progresses [30].

Laleman et al. (2023), in a systematic review of 10 randomized clinical trials, found no significant differences in MBL or PD between Ti and Zr abutments over five years, a finding consistent with the present study, which observed no significant differences in MBL at 12 months among Ti, Zr, and PMMA abutments [36]. Pesce et al. (2023) highlighted advantages of Zr in soft tissue health parameters, such as low BOP and PI compared to Ti. Despite these findings, the clinical parameters across all materials in this study remained consistent, suggesting the influence of patient-specific factors or variations in study design rather than the materials choice [37]. The aesthetic superiority of Zr abutments, especially for patients with thin gingival biotypes, is widely recognized; however, its potential drawbacks, including technical complications such as fractures, may limit its application in high-stress areas [36,38]. PMMA, while less extensively studied, demonstrates satisfactory performance without unique complications. This suggests it could serve as a cost-effective alternative for cases prioritizing aesthetics and affordability.

In our study, Periotest values were stable across all timepoints and groups. Factors that could influence Periotest values include the choice of abutment material, the rigidity of the crown, and the design of the suprastructure [39]. In the present study, Periotest measurements were consistently conducted using healing abutments across all groups to ensure standardization. Meta-analyses and studies have consistently concluded that there are minimal differences between Ti and Zr abutments in terms of MBL and biological complications, emphasizing instead the importance of surface characteristics, such as roughness and potential microgaps between implants and abutments influencing soft tissue outcomes. These findings align with the current results, supporting the notion that material composition plays a secondary role in determining long-term clinical outcomes.

These findings add to the growing evidence supporting the equivalence of various present abutment materials in maintaining peri-implant tissue health.

It is worth noting that all patients in this study were non-smokers, had no history of systemic diseases, and exhibited good periodontal health at baseline. Therefore, the results may not be generalizable to populations with higher baseline inflammation or systemic conditions that could exacerbate the inflammatory response to foreign materials.

An additional consideration in this study is the transition from gingiva formers to prosthetic restorations, which occurred three months after re-entry surgery in the Ti and Zr group. This procedural step may have had some influence on the peri-implant tissue response. Although efforts were made to ensure that the materials used for abutments and crowns adhered to the same manufacturing protocols and surface characteristics, the procedural change could introduce minor variability. While the materials were fabricated using the same protocols and no stains or glazing were applied in mucosa-contact areas, subtle factors such as differences in component geometry or handling may have affected soft tissue responses.

Furthermore, the small sample size and high variability in aMMP-8 levels, particularly in the zirconia and PMMA groups, may have limited the statistical power to detect more subtle differences between the materials. While the 12-month follow-up provides valuable insights into the peri-implant tissue response, a longer observation period may reveal more pronounced differences in bone remodeling and long-term clinical outcomes.

## 5. Conclusions

In conclusion, the results of this study suggest that the tested abutment material may have an impact on early peri-implant inflammatory responses; however, over time, aMMP-8 levels in peri-implant sulcular fluid decreased across all materials, and no significant differences were observed in clinical or radiographic outcomes after 12 months. All three materials—titanium, zirconium oxide, and PMMA—demonstrated high biocompatibility and supported healthy peri-implant tissue responses in the long term. Future research with larger sample sizes and inclusion of patients with systemic conditions or compromised periodontal health would help to further clarify the role of abutment material in peri-implant disease progression.

## Figures and Tables

**Table 1:** Timeline of visits and procedures for implant stability and peri-implant health monitoring, showing when X-rays, Periotest measurements, and aMMP-8 assessments were performed. “X” indicates the procedures conducted at each visit.

Visit	Time After		X-Ray	Periotest	aMMP-8
	Implant Surgery	Uncovering			
1	−7 Days				
2	0 Days		X	X	
3	1 Week				
4	1 Month				
5	2 Months				
6	3 Months	0 Days		X	
7	3.5 Months	2 Weeks			X
8	5 Months	2 Months			X
9	6 Months	3 Months			X
10	9 Months	6 Months			X
11	15 Months	12 Months	X	X	X

**Table 2:** aMMP-8 levels in implants and natural teeth across timepoints for different abutment materials.

Time After Uncovering	Group	Type	*N*	Min	Max	Mean	SD
2 Weeks	Titanium	Implant	10	5.2	599.0	168.0	199.6
		Natural tooth	10	58.8	513.8	299.9	193.2
	Zirconia	Implant	10	15.2	655.1	427.3	220.6
		Natural tooth	10	30.0	657.6	279.8	246.2
	PMMA	Implant	10	46.0	695.6	287.5	234.3
		Natural tooth	10	46.4	612.4	270.2	205.9
2 Months	Titanium	Implant	10	11.2	605.9	156.8	203.8
		Natural tooth	10	50.4	678.6	272.8	214.0
	Zirconia	Implant	10	16.0	645.8	314.5	294.0
		Natural tooth	10	71.6	664.9	318.6	236.2
	PMMA	Implant	9	9.6	648.1	226.5	208.6
		Natural tooth	10	0.0	236.8	121.6	73.3
3 Months	Titanium	Implant	10	10.0	216.0	79.0	69.1
		Natural tooth	10	38.0	634.8	306.5	218.7
	Zirconia	Implant	10	46.8	588.1	206.2	202.6
		Natural tooth	10	29.2	561.9	278.0	204.1
	PMMA	Implant	10	26.0	560.6	202.0	186.5
		Natural tooth	10	7.2	567.3	149.6	156.5
6 Months	Titanium	Implant	10	9.6	552.9	160.2	211.9
		Natural tooth	10	3.6	645.1	216.9	263.2
	Zirconia	Implant	10	3.6	624.7	216.1	244.5
		Natural tooth	10	0.4	618.3	236.5	238.9
	PMMA	Implant	10	6.8	640.0	291.0	256.9
		Natural tooth	10	17.2	539.0	175.8	178.9
12 Months	Titanium	Implant	10	0.0	693.5	164.8	249.7
		Natural tooth	10	6.0	562.6	151.1	216.8
	Zirconia	Implant	9	3.6	576.3	122.6	192.1
		Natural tooth	10	0.0	480.0	111.8	180.6
	PMMA	Implant	10	0.8	511.2	125.6	150.2
		Natural tooth	10	1.2	473.3	95.1	141.4

**Table 3:** Differences in aMMP-8 levels between implants and natural teeth at various timepoints, with significance indicated by an asterisk (*).

Time After Uncovering	Group	*N*	Min	Max	Mean	SD	*p*-Value	
2 weeks	Titanium	10	−158	459.4	131.9	222.9	*p* = 0.094	
	Zirconia	10	−424.5	141.2	−147.6	206.5	*p* = 0.050	*
	PMMA	10	−494.8	379.2	−17.3	297.1	*p* = 0.858	
2 months	Titanium	10	−555.5	617.8	116.0	302.0	*p* = 0.255	
	Zirconia	10	−529.1	527.2	4.2	374.7	*p* = 0.973	
	PMMA	9	−527.3	131.6	−91.5	208.9	*p* = 0.225	
3 months	Titanium	10	−10.4	509.6	227.4	207.5	*p* = 0.007	*
	Zirconia	10	−400.7	481.1	71.8	276.6	*p* = 0.433	
	PMMA	10	−451	372.5	−52.5	221.2	*p* = 0.473	
6 months	Titanium	10	−506.2	558	56.8	359.2	*p* = 0.629	
	Zirconia	9	−275.7	479.6	23.3	224.0	*p* = 0.768	
	PMMA	10	−514.4	44	−115.2	188.9	*p* = 0.086	
12 months	Titanium	10	−314.8	532.2	−13.6	226.7	*p* = 0.853	
	Zirconia	9	−246.4	358.8	1.6	158.4	*p* = 0.976	
	PMMA	10	−478.4	308.5	−30.6	195.4	*p* = 0.633	

## Data Availability

The data supporting the findings of this study are available from the corresponding author upon reasonable request.
